# Shared Decision-Making for Partial Oral Antibiotic Treatment of Infective Endocarditis: A Case Series

**DOI:** 10.1093/ofid/ofae166

**Published:** 2024-03-19

**Authors:** Sami El-Dalati, Marice Ruiz Conejo Castillo, Luke Strnad, Hassan Reda, Tessa London, Michael Sekela, Bobbi Jo Stoner

**Affiliations:** Division of Infectious Diseases, Department of Internal Medicine, University of Kentucky Medical Center, Lexington, Kentucky, USA; CrescentCare, New Orleans, Louisiana, USA; Division of Infectious Diseases, Department of Internal Medicine, Oregon Health and Science University, Portland, Oregon, USA; Department of Cardiac Surgery, University of Kentucky College of Medicine, Lexington, Kentucky, USA; Department of Cardiac Surgery, University of Kentucky College of Medicine, Lexington, Kentucky, USA; Department of Cardiac Surgery, University of Kentucky College of Medicine, Lexington, Kentucky, USA; University of Kentucky College of Pharmacy, Lexington, Kentucky, USA

**Keywords:** endocarditis, clinical outcomes, multidisciplinary team, oral antibiotic treatment, patients who inject drugs

## Abstract

Although literature has demonstrated the noninferiority of oral antibiotics in the treatment of infectious endocarditis, widespread adoption of this practice has yet to occur in the United States. We report on 32 patients with infectious endocarditis treated by a multidisciplinary endocarditis team and a standardized approach to partial oral antibiotic therapy with a high rate of clinical success.

Infective endocarditis (IE) is associated with significant morbidity and mortality as well as cost to the United States (US) healthcare system [1, 2]. In recent years, literature has demonstrated that partial oral antimicrobial therapy is noninferior to treatment regimens delivered entirely intravenously [3, 4]. Moreover, no published study has found that intravenous (IV) antibiotics are superior to oral antibiotics in treatment of IE [5]. While European IE guidelines have incorporated recommendations for oral antibiotics in certain cases, North American professional societies have yet to formally adopt this as an equivalent treatment strategy, except in cases of *Candida* endocarditis treated with oral fluconazole step-down therapy [6–8]. The studies that have emphasized the role of oral antibiotic therapy had very rigorous screening and follow-up requirements as well as relatively small numbers of persons who inject drugs (PWIDs). For example, Iversen et al performed a randomized controlled trial requiring patients assigned to oral treatment to follow-up on an outpatient basis 2–3 times per week, and all patients underwent transesophageal echocardiography 1–3 days prior to completion of therapy to rule out progression of disease. Their study only included 5 PWIDs out of 400 participants and was notably conducted in Denmark, which has a markedly different healthcare system and network of social services compared to the US [3]. Freling et al performed a retrospective cohort study in the US after implementing an expected practice and demonstrated similar outcomes for patients treated with partial oral versus IV therapy, but without the use of a multidisciplinary endocarditis team or details about how patients were selected for each treatment strategy [4]. Consequently, questions remain about the applicability of existing data to the PWID population and how to realistically implement oral therapy as part of clinical practice [9]. We describe our 1-year experience of treatment with oral antimicrobials at a tertiary medical center with high rates of drug use–associated IE, utilizing a multidisciplinary endocarditis team as well as an approach to treatment choice emphasizing shared decision-making and harm reduction [10].

## METHODS

Institutional review board exemption was obtained from the University of Kentucky. In September 2021, University of Kentucky Healthcare (UK) created a multidisciplinary endocarditis team (MDET) and cardiovascular infectious diseases (CVID) consult service. The MDET is comprised of providers from infectious diseases, cardiac surgery, cardiology, addiction medicine, neurosurgery, neurology, physical medicine and rehabilitation, palliative care, and ethics. The group formally meets weekly to discuss all inpatients with IE and document its recommendations in the electronic medical record. Frequent communication between MDET providers and clinical primary teams also occurs outside of scheduled meeting times. The CVID consult service is an interdisciplinary team housed in the Division of Infectious Diseases and comprises an attending physician, advanced practice provider, nurse navigator, pharmacist, and social worker. The CVID service consults on all inpatients with IE and follows them throughout their hospitalization and transition to outpatient. The CVID team coordinates the weekly MDET meetings and works with other specialties to coordinate follow-up and outpatient testing [11]. The MDET and CVID consult service developed an internal protocol to guide the use of partial oral antibiotic therapy in patients with IE (Supplementary Figure 1), which was derived from the procedure utilized by Iversen et al [3]. During this period, once an individual was deemed stable for discharge by the MDET and primary team, the CVID service presented the patient with the evidence for partial oral antimicrobial treatment in cases of IE caused by methicillin-susceptible *Staphylococcus aureus* (MSSA), *Enterococcus faecalis*, streptococci, and fluconazole-susceptible *Candida* species. Specifically, the CVID service explained that multiple studies have demonstrated similar outcomes for patients with the above organisms transitioned to oral therapy after approximately 10 days of IV treatment compared to full courses of IV antimicrobials. Copies of the referenced literature were made available to patients upon request. Oral treatment was offered to all patients with the above organisms, who were stable for discharge to home. This option was also presented to patients with substance use disorder and IE. Some patients, due to the acuity of their illness, were not clinically stable for discharge until well after they completed their IE antimicrobial treatment. Patients referred to acute or subacute rehabilitation facilities based on their physical and occupational therapy needs were offered oral antimicrobials if their anticipated rehabilitation stay was shorter than the expected duration of antimicrobial therapy.

If a patient preferred to receive a full course of IV treatment, they were then evaluated by the outpatient parenteral antibiotic therapy (OPAT) team. The OPAT group at UK predates the existence of the MDET and CVID service and does not routinely attend MDET meetings. Once a referral is made to the OPAT team, the patient is then seen by an OPAT nurse navigator who performs an evaluation of the patient's social situation and ability to perform infusions either independently or with the assistance of a friend/family member. Particular attention is paid to whether the patient has a place of residence where medication can be safely delivered; electricity and refrigeration to store specific antimicrobials; a working telephone to communicate regarding adverse medication events, laboratory abnormalities, or other complications of treatment; access to home health or another clinic/facility that can perform weekly laboratory studies and peripherally inserted central catheter dressing changes; and transportation to follow-up appointments. Substance use is not considered an absolute contraindication to enrolling in OPAT, but depending on the infectious diseases provider it may lead to the patient being declined. Based on these factors, the OPAT team makes a final assessment regarding whether the patient can discharge home to receive IV antimicrobials. Patients discharged to lower-acuity medical facilities are still enrolled in OPAT for the purposes of laboratory monitoring but do not undergo a nurse navigator evaluation.

Using this shared decision-making approach (summarized in Supplementary Figure 2), the CVID service and patients collaborated on whether to complete antibiotic therapy with oral or IV regimens. Individuals who left the hospital before medically advised (BMA), regardless of the organism, were also offered oral antimicrobial treatment. Whenever possible, bedside medication delivery from the hospital's outpatient pharmacy was performed. In some instances, patients would depart their rooms before their medication could be delivered. In those cases, their oral antibiotics were sent to their preferred ambulatory pharmacy. Patients were scheduled for infectious diseases follow-up within 2 weeks of discharge. At these appointments, laboratory monitoring with complete blood counts and comprehensive metabolic panels was performed. Blood cultures were obtained if patients reported symptoms concerning for recurrent bloodstream infection.

**Figure 1.**
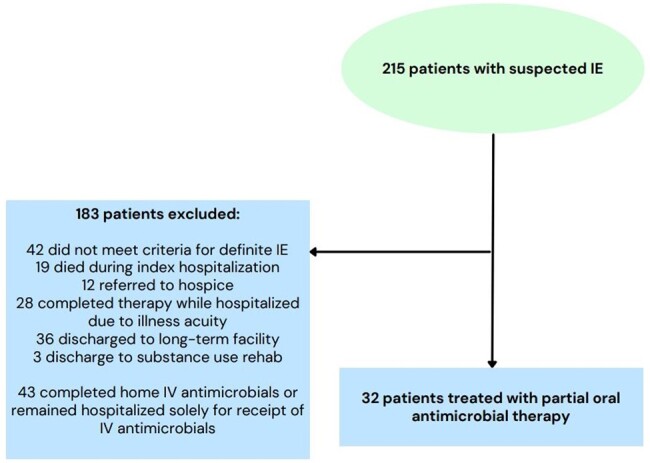
Study enrollment flowchart for patients with infective endocarditis treated with partial oral antimicrobial therapy. Abbreviations: IE, infective endocarditis; IV, intravenous.

The authors then performed a retrospective cohort study of patients admitted to UK with suspected or confirmed IE. Institutional review board approval was obtained from UK. Cases were identified from the institutional MDET registry. Patients were excluded from this analysis if they were deemed to not have IE by the MDET, died during the index admission, or completed IV antibiotic therapy while inpatient, at a long-term treatment facility, or at home (Figure 1). Individual chart review was performed by study investigators, and demographic, clinical, and outcome variables were recorded. The reason(s) for transitioning to oral antimicrobial therapy were also recorded. The primary outcome was a composite of relapsed infection and mortality at 90 days. Relapsed infection was defined as a positive culture from any site with the same microorganism identified during the index hospitalization. For patients who did not attend follow-up appointments, data regarding relapsed infection and mortality were obtained from other encounters in the electronic medical record.

## RESULTS

### Demographics

Between 7 September 2021 and 6 September 2022, 32 patients with definite IE confirmed by the MDET and treated with partial antimicrobial therapy were identified. The median age was 42 years (interquartile range [IQR], 34.8–49.3) and 40.6% were female (Table 1). Twenty-nine (90.6%) patients were White, 2 (6.3%) were Latinx, and 1 (3.1%) was African American. The median Charlson Comorbidity Index score was 1 (IQR, 0–2.5). Twenty-two (68.9%) patients had history of injection drug use and the majority (53.1%) had comorbid hepatitis B and/or C. Eight (25%) patients had a history of prior IE and 5 (15.6%) individuals had prosthetic valves.

**Table 1.** Demographic and Clinical Variables and Treatment Outcomes for Patients Receiving Partial Oral Antimicrobial Therapy for Infective Endocarditis (N = 32)

Variable	No. (%)
Demographic variables	
Age, y, median (IQR)	42 (34.8–49.3)
Female sex	40.6 (13)
Race/ethnicity	
White	90.6 (29)
African American	3.1 (1)
Latinx	6.3 (2)
Charlson Comorbidity Index score, median (IQR)	1 (0–2.5)
Injection drug use	68.9 (22)
Clinical variables	
MSSA	34.4 (11)
MRSA	3.1 (1)
Streptococci	15.6 (5)
*Enterococcus faecalis*	21.9 (7)
Polymicrobial	21.9 (7)
Culture-negative	3.1 (1)
Aortic valve	21.9 (7)
Mitral valve	12.5 (4)
Tricuspid	53.1 (17)
Multiple valves	12.5 (4)
CIED	3.1 (1)
Prosthetic valve	15.6 (5)
Prior infective endocarditis	25 (8)
Hepatitis B	15.6 (5)
Hepatitis C	37.5 (12)
Septic emboli	56.3 (18)
Septic arthritis/osteomyelitis	28.1 (9)
Percutaneous mechanical aspiration	9.4 (3)
Valve surgery	40.6 (13)
Clinical outcomes	
Duration of IV therapy, d, median (IQR)	18 (10–26)
Duration of oral therapy, d, median (IQR)	18 (11–25)
Patients receiving consolidation or suppressive therapy	18.8 (6)
Completed oral therapy	78.1 (25)
30-d readmission	9.4 (3)
90-d mortality	3.1 (1)
Relapsed infection	3.1 (1)

Data are presented as No. (%) unless otherwise indicated.

Abbreviations: CIED, cardiac implantable electronic device; IQR, interquartile range; IV, intravenous; MRSA, methicillin-resistant *Staphylococcus aureus*; MSSA, methicillin-susceptible *Staphylococcus aureus*.

### Index Infection

*Staphylococcus aureus* was the most commonly isolated pathogen, in 12 (37.5%) patients. MSSA was identified in 11 patients and methicillin-resistant *S aureus* (MRSA) in 1. *Enterococcus faecalis* was seen in 7 (21.9%) cases, and streptococcal species were cultured in 5 cases (15.6%). Seven (21.9%) patients had polymicrobial infections, including 3 with *Candida* species. Only 1 case had negative blood cultures. This individual met criteria for definite IE and had positive wound cultures for a *Streptococcus* species. More than half of patients in the series (53.1%) had isolated native tricuspid valve IE. Eleven patients (34.4%) had isolated left-sided endocarditis. The remaining 4 (12.5%) cases involved multiple valves. Eighteen (56.3%) patients had septic emboli and 9 (28.1%) had concomitant osteoarticular infections. Thirteen (40.6%) underwent surgical valve repair/replacement during the index hospitalization and 3 (9.4%) were treated with percutaneous mechanical aspiration of a tricuspid valve vegetation.

### Antimicrobial Therapy

The majority of patients (75%) were treated with multiple antimicrobials, including 68.9% of cases treated with 2 agents (Supplementary Table 1). Linezolid was the most commonly prescribed antibiotic, in 65.6% of patients. This was followed by cefadroxil (38.1%), fluoroquinolones (31.3%), and amoxicillin (28.1%).

Eight patients (25%) were treated with a single oral agent. Of these, 2 patients were transitioned to oral doxycycline after receiving >5 weeks of IV therapy. Both of these individuals discharged rapidly BMA and before prior authorizations could be obtained for the protocol's oral antibiotics. Two patients were initially discharged receiving IV dalbavancin and developed side effects requiring a transition to oral linezolid. One patient, the only case of culture-negative IE, was treated with both IV dalbavancin and oral levofloxacin. One patient with *Candida* IE was transitioned to monotherapy with isavuconazole. One patient with MSSA tricuspid valve endocarditis was treated with linezolid alone after 3 weeks of IV therapy.

### Reasons for Selecting Oral Therapy

Twenty-two individuals (68.9%) transitioned to partial oral treatment of their IE as a result of shared decision-making with the CVID service (Supplementary Table 2). Seven patients were provided with oral antimicrobials at the time of BMA discharge. Two patients experienced OPAT complications that required transition to an oral regimen, and 1 patient was declined by the OPAT team.

### Clinical Outcomes

The median duration of IV therapy was 18 days (IQR:10–26 days) and the median duration of oral antimicrobial therapy was an additional 18 days (IQR:11–25 days). Six patients were transitioned to a prolonged course of consolidative or suppressive oral antimicrobials. This was done in patients with *S aureus* vertebral osteomyelitis, in patients with medically treated prosthetic valve endocarditis caused by *E faecalis*, and surgically treated patients with positive valve cultures for *Candida* species [12–14]. Twenty-five patients (78.1%) were confirmed to have completed oral antimicrobials at outpatient follow-up (Table 1). Patients were documented as completing therapy if they attended outpatient follow-up and verbally reported taking their course of oral antimicrobials. Patients were marked as not completed if they were lost to follow-up or did not pick up prescribed refills. The composite outcome of 90-day mortality and relapsed infection occurred in 2 patients (6.3%). The patient who passed away had severe aortic regurgitation as well as cirrhosis and transitioned to hospice after they were deemed prohibitively high-risk for valve replacement. The case of relapsed infection occurred in a patient who self-discontinued oral suppressive therapy approximately 7 weeks after the initial course of treatment.

The secondary outcome of 30-day hospital readmission occurred in 3 patients (9.4%). All were readmitted for heart failure–related complications, including 1 patient with a noninfectious left-sided pleural effusion.

## DISCUSSION

In this retrospective case series, we report 32 patients with IE treated with partial oral antimicrobial therapy guided by an institutional standardized workflow. The primary outcomes of relapsed infection or mortality at 90 days occurred in 2 patients, for a treatment failure rate of 6.3%. Additionally, there were low rates of readmission, with no patients requiring readmission due to adverse events from oral therapy or progressive infection. More than two-thirds of patients in our study had a history of injection drug use, suggesting that PWIDs with IE can successfully complete oral antimicrobial treatment.

Our study is unique in 2 notable ways. First, the majority of patients were carefully selected for oral treatment by both a multidisciplinary team and a CVID consult service. Second, the decision to transition to oral antimicrobials was made utilizing a standardized workflow and shared decision-making between patients and CVID providers rather than leaving the decision primarily to heterogenous physicians or clinical randomization. While individuals leaving BMA were also offered antimicrobials, they only comprised 21.9% of the study population. These findings highlight that a subset of patients may prefer oral antimicrobial to IV therapy when presented with a standardized opportunity to make an informed decision. However, many patients stable for discharge (n = 43) did still select to complete treatment with IV antimicrobials. Anecdotally, the authors noted that many patients still perceived a superiority of IV treatment even after being presented with evidence to the contrary.

The median duration of total antibiotic therapy was 36 days. While this is shorter than the often utilized 6-week regimen, our study included patients with native valve streptococcal IE and right-sided MSSA IE, which consensus United States and European guidelines recommend treating for durations <6 weeks [6, 7]. Given the limited data supporting the efficacy of 6-week regimens, these results support the use of shorter durations of therapy with specific organisms. Additionally, 25% of patients were treated with a single agent, suggesting that a subset of patients may not require dual antimicrobial therapy to effectively eradicate their infection.

Our study is limited by its retrospective nature, the lack of a control group treated with IV antibiotics alone, and the low number of patients with MRSA. It is also possible that patients could have had other metastatic complications or progression of their infection that was not captured by defining relapsed infection by the presence of a positive culture. However, previous studies have demonstrated treatment failure rates of approximately 12%–18% for patients receiving IV therapy for IE [3, 4, 15]. In the absence of data demonstrating the superiority of IV antibiotics, the challenges PWIDs face obtaining OPAT access, and the potential complications of OPAT generally, providers can consider step-down to oral therapy in select patients with IE who are clinically stable for discharge [16, 17]. A multidisciplinary endocarditis team may better select individuals for this transition, and a standardized workflow for offering all eligible patients oral therapy may increase the uptake of this intervention as evidenced by the fact that we treated 32 patients with oral therapy in 12 months (2.7/month) compared to Freling et al where 46 individuals received oral therapy over 42 months (1.1/month). Further data are required about the ideal antimicrobial regimens and duration of therapy as well as the most optimized way to integrate a multidisciplinary endocarditis team in the oral therapy decision-making process.

## Supplementary Material

ofae166_Supplementary_Data

## Supplementary Data

Supplementary materials are available at *Open Forum Infectious Diseases* online. Consisting of data provided by the authors to benefit the reader, the posted materials are not copyedited and are the sole responsibility of the authors, so questions or comments should be addressed to the corresponding author.

## Notes

***Patient consent.*** Institutional review board exemption was obtained from the University of Kentucky, and patient consent was not required.
