# Supplementary material for: Shared Decision-Making for Partial Oral Antibiotic Treatment of Infective Endocarditis: A Case Series
Source: Open Forum Infect Dis. 2024 Mar 19;11(4):ofae166. doi: 10.1093/ofid/ofae166 (PMC10996124; doi:10.1093/ofid/ofae166)
Supplement: ofae166_Supplementary_Data [file ofae166_supplementary_data.zip › Partial Oral Antibiotic Treatment Supplemental Figure 1.pdf]

Supplemental Figure 1. University of Kentucky Healthcare Endocarditis Oral Antibiotic Therapy

PLEASE TAKE NOTE: The purpose of this guide (not guideline) is to help **experienced infectious diseases (ID) physicians and pharmacists** better assess all potential treatment options for each specific patient. It is still of the ID clinician's expertise and judgement of the individual patient scenario to determine whether a specific oral regimen is appropriate versus intravenous therapy. The guide below assumes pathogen identification and sensitivity to the respective antibiotics suggested. It remains essential to assess the patient's potential adherence to the oral regimen prior to recommending the switch from intravenous therapy. **This guidance document is not recommended for use outside of the MDET/Infectious Disease consult service.**

Oral Antibiotic Recommendations

****For all recommended antimicrobials only utilize if there has been confirmed in vitro susceptibility of the organism to the agent**

**Pathogen Specific**	**Diagnosis (Modifying Factors)**	**Targeted Partial Oral Antibiotic Therapy**	**Comments**
Methicillin Susceptible *Staphylococcus aureus*	Endocarditis (Native or Prosthetic Valve)	Linezolid[1] 600 g Q12H<br>AND<br>Cefadroxil 1 g Q12H<br><br>OR<br><br>Linezolid[1] 600 g Q12H<br>AND<br>Dicloxacillin 1 g Q6H<br><br>OR<br><br>Linezolid[1] 600 g Q12H<br>AND<br>Rifampin[2] 600 mg Q12H<br><br>OR	Consider in patients who meet the following criteria:<br><br>- Completed ≥ 10 days of IV antibiotic therapy from the 1st negative blood culture<br>- Completed ≥ 7 days of IV antibiotic therapy from the operative date<br>- Afebrile >48 hours<br>- WBC count <15,000<br>- CRP <2.0 (ref range <0.8) or 25% of value at diagnosis<br>- BMI <40<br>- Can tolerate PO medications
Methicillin Susceptible Coagulase Negative Staphylococci			

Infectious Diseases/MDET use ONLY

Pathogen Specific	Diagnosis (Modifying Factors)	Targeted Partial Oral Antibiotic Therapy	Comments
		Linezolid[1] 600 mg Q12H<br>AND<br>Rifampin[2] 600 mg Q12H<br><br>Duration: Refer to UK guidelines	
Methicillin Resistant *Staphylococcus aureus*	Endocarditis (Native and Prosthetic valve)	N/A	There is insufficient data to support the use of targeted partial oral antibiotic therapy for methicillin resistant *Staphylococcal aureus* endocarditis.
Methicillin Resistant Coagulase Negative Staphylococci	Endocarditis (Native and Prosthetic Valve)	Linezolid[1] 600 mg Q12H<br>AND<br>Rifampin[2] 600 mg Q12H<br><br>Duration: Refer to UK guidelines	Consider in the following patients:<br><br>- Completed ≥ 10 days of IV antibiotic therapy from the 1st negative blood culture<br>- Completed ≥ 7 days of IV antibiotic therapy from the operative date<br>- Afebrile >48 hours<br>- WBC count <15,000<br>- CRP <2.0 mg/dL (ref range <0.8) or 25% of value at diagnosis<br>- BMI <40<br>- Can tolerate PO medications
Ampicillin Susceptible *Enterococcus faecalis*	Endocarditis (Native and Prosthetic valve)	Amoxicillin 1 g Q6H<br>AND<br>Linezolid[1] 600 mg Q12H<br><br>OR	Consider in the following patients<br><br>- Completed ≥ 10 days of IV antibiotic therapy from the 1st negative blood culture<br>- Completed ≥ 7 days of IV antibiotic therapy from the operative date (if surgically managed)

Infectious Diseases/MDET use ONLY

Pathogen Specific	Diagnosis (Modifying Factors)	Targeted Partial Oral Antibiotic Therapy	Comments
		Levofloxacin[3] 750 mg Q24H<br>AND<br>Amoxicillin 1 g Q6H<br><br>OR<br><br>Linezolid[1] 600 mg Q12H<br>AND<br>Levofloxacin[3] 750 mg Q24H<br><br>Duration: Refer to UK Guidelines	- Afebrile >48 hours<br>- WBC count <15,000<br>- CRP <2.0 mg/dL (ref range <0.8) or 25% of value at diagnosis<br>- BMI <40<br>- Can tolerate PO therapy
Highly penicillin susceptible viridans group streptococci (VGS), *Streptococcus gallolyticus (bovis),* other streptococci with an MIC to penicillin ≤ 0.12mcg/ml	Endocarditis (Native and Prosthetic Valve)	Amoxicillin 1 g Q6H<br>AND<br>Linezolid[1] 600 mg Q12H<br><br>OR<br><br>Linezolid[1] 600 mg Q12H<br>AND<br>Levofloxacin[3] 750 mg Q24H<br><br>Duration: Refer to UK guidelines	Consider in the following patients:<br><br>- Completed ≥ 10 days of IV antibiotic therapy from the 1st negative blood culture<br>- Completed ≥ 7 days of IV antibiotic therapy from the operative date (if surgically managed)<br>- Afebrile >48 hours<br>- WBC count <15,000<br>- CRP <2.0 mg/dL (ref range <0.8) or 25% of value at diagnosis<br>- BMI <40<br>- Can tolerate PO therapy<br><br>*Substitute Cefadroxil 1 gm twice a day for amoxicillin in penicillin allergic patients
VGS and *Streptococcus gallolyticus*	Endocarditis (Native and Prosthetic Valve)	Linezolid[1] 600 mg Q12H<br>AND<br>Amoxicillin 1 g Q6H	Consider in the following patients:<br><br>- Completed ≥ 10 days of IV antibiotic therapy from the 1st negative blood culture

Infectious Diseases/MDET use ONLY

Pathogen Specific	Diagnosis (Modifying Factors)	Targeted Partial Oral Antibiotic Therapy	Comments
(bovis) with penicillin MIC of >0.12		OR<br><br>Linezolid[1] 600 mg Q12H<br>AND<br>Levofloxacin[3] 750 mg Q24H<br><br>OR<br><br>Amoxicillin 1 g Q6H<br>AND<br>Levofloxacin[3] 750 mg Q24H<br><br>OR<br><br>Linezolid[1] 600 mg Q12H<br>AND<br>Rifampin[2] 600 mg Q12H<br><br>Duration: Refer to UK guidelines	- Completed ≥ 7 days of IV antibiotic therapy from the operative date (if surgically managed)<br>- Afebrile >48 hours<br>- WBC count <15,000<br>- CRP <2.0 mg/dL (ref range <0.8) or 25% of value at diagnosis<br>- BMI <40<br>- Can tolerate PO therapy

1) Use **Linezolid** with caution in patients on concurrent methadone as methadone may enhance the serotonergic effect of Monoamine Oxidase Inhibitors and could result in serotonin syndrome. CBC with differential should be monitored weekly for patients discharged on linezolid, especially when used for durations > 14 days.

2) **Rifampin** may induce the metabolism of many medications, including methadone, warfarin and other oral anticoagulants, resulting in decreased drug levels. Recommend initiation of therapy while inpatient and monitoring need for potential methadone dose adjustments.

3) All patients receiving **Levofloxacin** should have a documented QTc ≤500 ms. Use with caution in patients with methadone as both medications can prolong the QTc. Please use additional caution with initiation of any other new QT prolonging medications.

Infectious Diseases/MDET use ONLY

Antimicrobial Monitoring and Drug Interactions

Antibiotic	Lab Monitoring	Renal Dosing Adjustment	Drug-Drug Interactions
Cefadroxil	Not routinely indicated, consider CBC and CMP every 2 weeks	Yes	N/A
Dicloxacillin	Not routinely indicated, consider CBC and CMP every 2 weeks	No	N/A
Levofloxacin	Every 1-2 weeks serum BUN, creatinine	No	Use with caution with other QT prolonging medications<br>Examples: methadone, amiodarone, macrolides, azole antifungal, SSRIs, antipsychotics
Linezolid	Weekly CBC with differential for courses >14 days	No (Use with renal impairment may increase the risk of thrombocytopenia)	Use with caution in patients on concurrent methadone or other serotonergic medications as they may enhance the serotonergic effect of Linezolid and could result in serotonin syndrome.
Rifampin	LFTs every 2 weeks	No	Potent CYP3A4 inducer which may result in decreased drug concentration of concurrently prescribed medications<br>Examples: Methadone, Warfarin, DOACs, azole antifungals, ticagrelor

Infectious Diseases/MOET use ONLY
