# Supplementary material for: Shared Decision-Making for Partial Oral Antibiotic Treatment of Infective Endocarditis: A Case Series
Source: Open Forum Infect Dis. 2024 Mar 19;11(4):ofae166. doi: 10.1093/ofid/ofae166 (PMC10996124; doi:10.1093/ofid/ofae166)
Supplement: ofae166_Supplementary_Data [file ofae166_supplementary_data.zip › Partial Oral Antibiotic Treatment Supplementary Table 1.docx]

Supplemental Table 1. Oral antimicrobials utilized for treatment of infective endocarditis.

| **Regimen/Agent** | **N = 32** |
| --- | --- |
| Combination Regimen, % (n) | 75 (24) |
| Two Drug Regimen, % (n) | 68.9 (22) |
| Monotherapy, % (n) | 25 (8) |
| Linezolid, % (n) | 65.6 (21) |
| Amoxicillin, % (n) | 28.1 (9) |
| Cefadroxil, % (n) | 38.1 (8) |
| Fluoroquinolone, % (n) | 31.3 (10) |
| Rifampin, % (n) | 9.4 (3) |
| Doxycycline, % (n) | 6.3 (2) |
