# Supplementary material for: Shared Decision-Making for Partial Oral Antibiotic Treatment of Infective Endocarditis: A Case Series
Source: Open Forum Infect Dis. 2024 Mar 19;11(4):ofae166. doi: 10.1093/ofid/ofae166 (PMC10996124; doi:10.1093/ofid/ofae166)
Supplement: ofae166_Supplementary_Data [file ofae166_supplementary_data.zip › Partial Oral Antibiotic Treatment Supplementary Table 2.docx]

Supplemental Table 2. Reasons for pursuing oral antimicrobial therapy of infective endocarditis.

| **Reason for pursuing oral antimicrobial therapy** | N = 32 |
| --- | --- |
| Shared Decision Making, % (n) | 68.9 (22) |
| Discharge Before Medically Advised, % (n) | 21.9 (7) |
| Declined by OPAT, % (n) | 3.1 (1) |
| OPAT Complication, % (n) | 6.3 (2) |

Abbreviations: OPAT = Outpatient Parenteral Antimicrobial Therapy
