# Supplementary figures and images for: Shared Decision-Making for Partial Oral Antibiotic Treatment of Infective Endocarditis: A Case Series
Source: Open Forum Infect Dis. 2024 Mar 19;11(4):ofae166. doi: 10.1093/ofid/ofae166 (PMC10996124; doi:10.1093/ofid/ofae166)

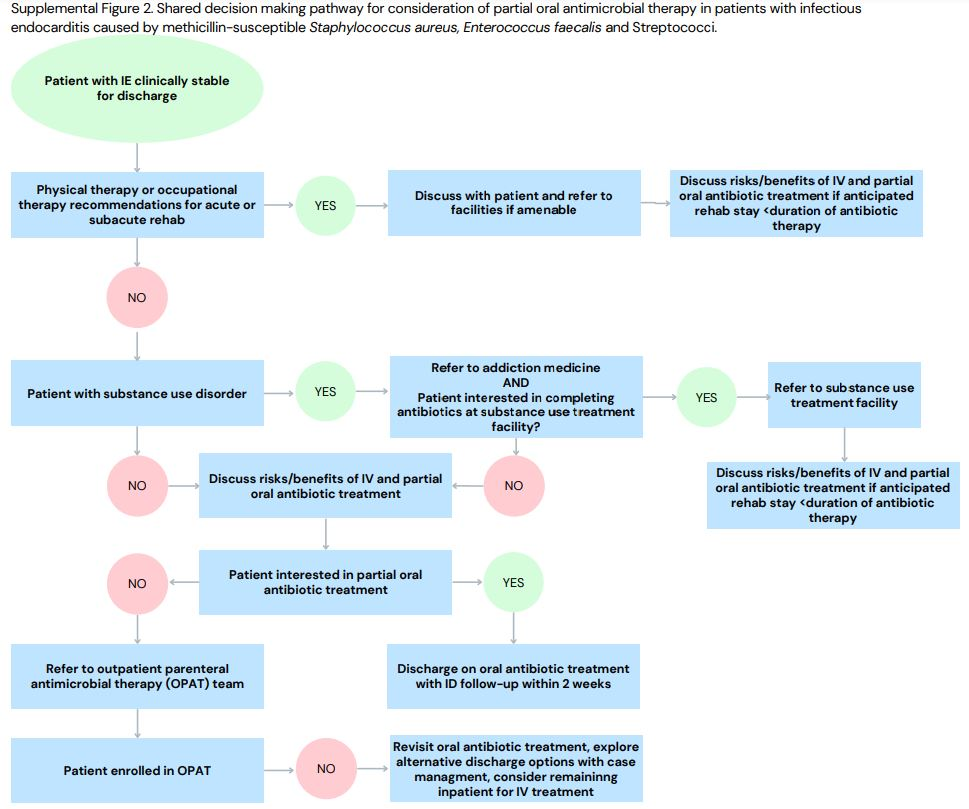

Supplement: ofae166_Supplementary_Data [file ofae166_supplementary_data.zip › Partial Oral Antibiotic Therapy Supplementary Figure 2 (3).tiff]
